# *Wnt1* Promotes Cementum and Alveolar Bone Growth in a Time-Dependent Manner

**DOI:** 10.1177/00220345211012386

**Published:** 2021-05-19

**Authors:** C. Nottmeier, N. Liao, A. Simon, M.G. Decker, J. Luther, M. Schweizer, T. Yorgan, M. Kaucka, E. Bockamp, B. Kahl-Nieke, M. Amling, T. Schinke, J. Petersen, T. Koehne

**Affiliations:** 1 Department of Orthodontics, University Medical Center Hamburg, Hamburg, Germany; 2 Department of Orthodontics, University of Leipzig Medical Center, Leipzig, Germany; 3 Department of Orthodontics, College of Stomatology, North China University of Science and Technology, Tangshan, China; 4 Department of Osteology and Biomechanics, University Medical Center Hamburg, Hamburg, Germany; 5 ZMNH, University Medical Center Hamburg-Eppendorf, Hamburg, Germany; 6 Max Planck Institute for Evolutionary Biology, Plön, Germany; 7 Institute for Translational Immunology and Research Center for Immunotherapy, University Medical Center, Johannes Gutenberg University, Mainz, Germany

**Keywords:** cementogenesis, mineralized tissue/development, signal transduction, periodontal ligament (PDL), Wnt/β-catenin signaling, bone biology

## Abstract

The WNT/β-catenin signaling pathway plays a central role in the biology of the periodontium, yet the function of specific extracellular WNT ligands remains poorly understood. By using a *Wnt1-*inducible transgenic mouse model targeting *Col1a1*-expressing alveolar osteoblasts, odontoblasts, and cementoblasts, we demonstrate that the WNT ligand WNT1 is a strong promoter of cementum and alveolar bone formation in vivo. We induced *Wnt1* expression for 1, 3, or 9 wk in Wnt1Tg mice and analyzed them at the age of 6 wk and 12 wk. Micro–computed tomography (CT) analyses of the mandibles revealed a 1.8-fold increased bone volume after 1 and 3 wk of *Wnt1* expression and a 3-fold increased bone volume after 9 wk of *Wnt1* expression compared to controls. In addition, the alveolar ridges were higher in Wnt1Tg mice as compared to controls. Nondecalcified histology demonstrated increased acellular cementum thickness and cellular cementum volume after 3 and 9 wk of *Wnt1* expression. However, 9 wk of *Wnt1* expression was also associated with periodontal breakdown and ectopic mineralization of the pulp. The composition of this ectopic matrix was comparable to those of cellular cementum as demonstrated by quantitative backscattered electron imaging and immunohistochemistry for noncollagenous proteins. Our analyses of 52-wk-old mice after 9 wk of *Wnt1* expression revealed that *Wnt1* expression affects mandibular bone and growing incisors but not molar teeth, indicating that *Wnt1* influences only growing tissues. To further investigate the effect of *Wnt1* on cementoblasts, we stably transfected the cementoblast cell line (OCCM-30) with a vector expressing *Wnt1*-HA and performed proliferation as well as differentiation experiments. These experiments demonstrated that *Wnt1* promotes proliferation but not differentiation of cementoblasts. Taken together, our findings identify, for the first time, *Wnt1* as a critical regulator of alveolar bone and cementum formation, as well as provide important insights for harnessing the WNT signal pathway in regenerative dentistry.

## Introduction

The WNT β-catenin signaling pathway is central for differentiation and proliferation of dental stem cells. The canonical WNT signaling is mediated via the binding of a WNT ligand to the 7-pass transmembrane receptor Frizzled (FZD) and the single-pass low-density lipoprotein receptor–related protein 5 or 6 (LRP5/6), leading to dephosphorylation-mediated stabilization and nuclear translocation of β-catenin (MacDonald and He 2012). Transgenic mouse studies have demonstrated that tissue-specific deletion or overexpression of β-catenin affects all stages of dental development, including root formation (Jarvinen et al. 2006; Liu et al. 2008; Chen et al. 2009; Kim et al. 2013; Zhang et al. 2013). However, until now, it has not been possible to target β-catenin pharmacologically (Nusse and Clevers 2017). A more promising strategy involves targeting extracellular components of the WNT β-catenin pathway. Here, an antibody targeting SOST, a negative regulator of WNT β-catenin, has only recently been approved for the treatment of women with severe postmenopausal osteoporosis (Cheng et al. 2020).

So far, mutations in *WNT10A* and *WNT10B* have been shown to cause syndromic tooth agenesis (in combination with malformation of other epithelial-derived organs such as hair and glands) and a nonsyndromic type of tooth agenesis in humans (Adaimy et al. 2007; Yu et al. 2016). In this study, we introduce the WNT ligand (WNT1) to be important for alveolar bone and cementum formation. Previously, we and others have shown that *Wnt1* is a central regulator of bone formation (Laine et al. 2013; Gori et al. 2016; Joeng et al. 2017; Luther et al. 2018). Mutations in *WNT1* are associated with osteogenesis imperfecta and with early onset osteoporosis (Fahiminiya et al. 2013; Keupp et al. 2013; Joeng et al. 2014), which is phenocopied in mice carrying the human *WNT1* mutation or with a cell-specific deletion of *Wnt1* in the osteoblast lineage. Furthermore, using a *Wnt1*-transgenic mouse line that expresses *Wnt1* under the control of a *Col1a1*-responsive promoter, it has been shown that *Wnt1* has a strong bone-anabolic function (Luther et al. 2018). Here, however, it remains to be determined whether *Wnt1* has the same bone-anabolic function in the alveolar bone. Moreover, since *Wnt1* is a key regulator of neural crest cells, which account for the majority of dental mesenchymal stem cells (Echelard et al. 1994), it is important to understand the role of *Wnt1* during postnatal tooth formation. We, therefore, asked how *Wnt1* affects postnatal dental development using *Wnt1*-transgenic mice with inducible expression of *Wnt1* in *Col1a1*-expressing cells such as alveolar osteoblasts, cementoblasts, and odontoblasts.

## Materials and Methods

### Mice

To generate Wnt1Tg mice, we crossed *Col1a1*-rtTA mice (Peng et al. 2008) with ptet-*Wnt1* mice (Gunther et al. 2003) on a mixed background. For genotyping, tail biopsies were used to identify double transgenic mice (Wnt1Tg) and control mice (mice lacking either *Col1a1*-rtTA or ptet-*Wnt1*). All other genotype litters were excluded from the study. We analyzed male mice at the age of 6 and 12 wk and female mice at the age of 52 wk. For each experiment including statistical analysis, a minimum of biological triplicates was used. Mice were allocated randomly to DOX-ON, DOX-OFF groups (coin flip), and experiments (micro–computed tomography [CT] and staining) were performed in a blinded fashion.

All mice were maintained in the animal facility of the University Medical Center Hamburg-Eppendorf in agreement with our animal ethics committee and comply with the ARRIVE Checklist (Percie du Sert et al. 2020) (see more details in the Appendix).

### Micro-CT and Stereomicroscopy

Skulls were analyzed by contact radiography with a Faxitron X-ray cabinet (Faxitron X-ray Corp.), followed by micro-CT scanning (µCT-40; Scanco Medical) with a voxel size of 15 μm, 55 kV, 145 µA, and 200 ms as previously described (Koehne et al. 2013) and analyzed (see the Appendix). For morphological analysis, incisors and mandibular molars were imaged with a high-resolution inverted microscope (Olympus DSX500i) and a 10× objective lens (DSX-10×; Leica Microsystems).

### Histology

Histology was performed on undecalcified specimens for histomorphometry and decalcified material for hematoxylin and eosin staining, as described in the Appendix.

### Immunohistochemistry

Immunohistochemistry was performed on 5-µm paraffin sections, as described in the Appendix.

### Fluorescence In Situ Hybridization (HCR 3.0)

Staining was performed, using the Molecular Instruments HCR v.3.0 protocol, for “generic sample on slide.” Fluorescence in situ hybridization probes were designed and purchased from Molecular Instruments.

### Quantitative Backscattered Electron Imaging

The degree of tissue mineralization was measured by means of the backscattered electron intensity, as previously described (Koehne et al. 2013) (see the Appendix).

### Cell Culture

The cementoblast cell line (OCCM-30; D’Errico et al. 2000) was cultured in Dulbecco’s modified Eagle’s medium (DMEM) containing 10% fetal bovine serum (v/v; HyClone; Logan). Proliferation and differentiation assays were performed as described in the Appendix.

## Results

### *Wnt1* Overexpression Affects Mandibular and Alveolar Bone Formation

**Figure 1.**
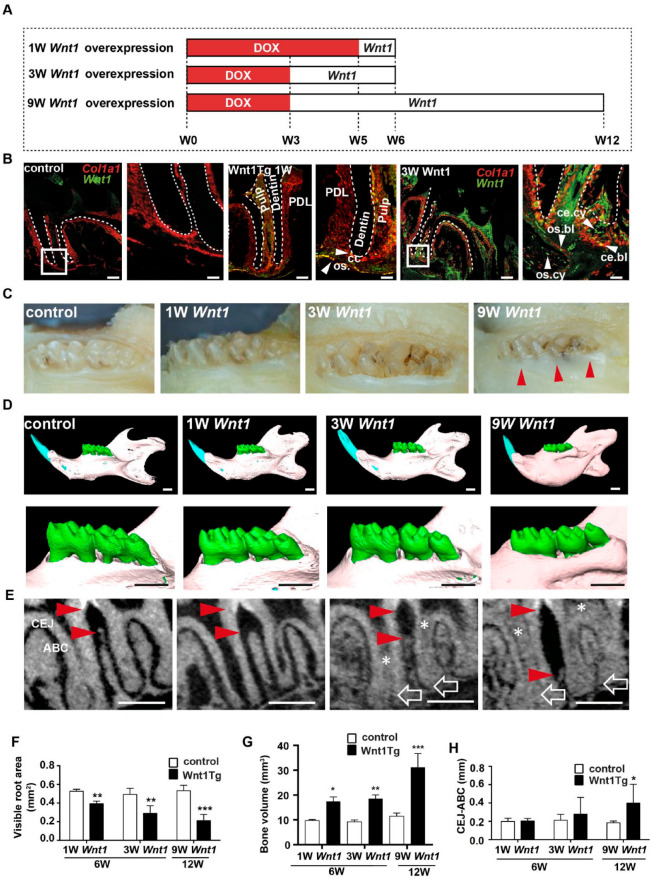
Conditional and tissue-specific expression of *Wnt1* affects alveolar bone formation. (**A**) Schematic representation of the feeding regime. Six-week-old mice were deprived of doxycycline 1 or 3 wk before sacrifice to induce short- and mid-term expression of *Wnt1*. Twelve-week-old mice were deprived of doxycycline 9 wk before sacrifice to induce long-term expression of *Wnt1*. (**B**) Undecalcified in situ hybridizations of molars of 6-wk-old wild-type (WT) and *Wnt1*-transgenic (Wnt1Tg) mice stained for *Col1a1* and *Wnt1* messenger RNA (mRNA). *Wnt1* was expressed for 1 wk (1W *Wnt1*) and 3 wk (3W *Wnt1*) in Wnt1Tg mice (ce.bl, cementoblast; ce.cy, cementocyte; os.bl, osteoblast; os.cy, osteocyte). Scale bars: 200 µm overview and 100 µm enlarged section. (**C**) Macroscopic images of molars of WT and Wnt1Tg mice. *Wnt1* was expressed for 1 wk (1W *Wnt1*) and 3 wk (3W *Wnt1*) in 6-wk-old mice and 9 wk (9W *Wnt1*) in 12-wk-old mice. The molars were partially covered by gingiva after 9 wk of *Wnt1* expression (black triangles). Scale bars: 1 mm. (**D**) Micro–computed tomography (CT) images of mandibles (upper panels) and mandibular molars (lower panels) from the same mice. The 3-dimensional images reveal overgrowth of alveolar bone in WntTg mice after 3 and 9 wk of *Wnt1* expression. Scale bars: 1 mm. (**E**) Micro-CT cross sections of the mandibular molars from the same mice. The bone loss in this area was measured as the distance between the cemento-enamel junction (CEJ) and the alveolar bone crest (ABC) (red triangles). Note the thick molar apices in Wnt1Tg mice (open white arrows) as well as the calcified tissue within the pulp (white asterisk). Scale bars: 0.5 mm. (**F–H**) Micro-CT quantification of visible root area (F), mandibular bone volume (G), and CEJ-ABC distance (H) from WT and Wnt1Tg mice at 6 and 12 wk of age (*n* = 4–7). *P < 0.05. **P < 0.01. ***P < 0.001.

To analyze short- and long-term effects of *Wnt1* expression on periodontal homeostasis, we used an inducible *Wnt1* transgenic mouse model (hereafter called Wnt1Tg), which targets alveolar osteoblasts and odontoblasts. In this model, doxycycline (DOX)–dependent *Wnt1* transgene expression is governed by the *Col1a1* promoter-driven tetracycline-controlled transcriptional activator (*Col1a1-tTA*), resulting in conditional *Wnt1* expression in cells expressing *Col1a1* (under the 2.3-kb fragment promoter) (Rossert et al. 1995; Peng et al. 2008) upon DOX withdrawal. We induced *Wnt1* expression for 1, 3, or 9 wk in Wnt1Tg mice and analyzed them at the age of 6 or 12 wk (Fig. 1A). To confirm transgene induction, we first compared the expression of *Wnt1* and *Col1a1* messenger RNA (mRNA) in molars and incisors of 6-wk-old Wnt1Tg mice and controls 1 and 3 wk after DOX withdrawal using in situ hybridization (Fig. 1B and Appendix Fig. 1B, C). Whereas the expression of *Wnt1* was not detectable in teeth sections of control mice (Fig. 1B and Appendix Fig. 1B), *Wnt1* mRNA expression was increased upon DOX withdrawal in osteoblasts, osteocytes, odontoblasts, cementoblasts, and cementocytes of Wnt1Tg mice (Fig. 1B and Appendix Fig. 1C).

Next, our initial macroscopic analysis of the teeth showed clear differences between Wnt1Tg and control mice (Fig. 1C and Appendix Fig. 1A). Here, the incisors of Wnt1Tg mice had a chalky white appearance after 3 and 9 wk of *Wnt1* expression (Appendix Fig. 1A), whereas the crowns of the mandibular molars were normally formed. However, the visible crown height appeared shorter in Wnt1Tg mice after 9 wk of *Wnt1* expression as compared to those of controls (Fig. 1C).

We next took advantage of micro-CT to analyze the mandibular molars (M1–M3) of Wnt1Tg mice, in which doxycycline has been removed for 1, 3, or 9 wk. The 3-dimensional (3D) segmentation images revealed a clear increase of mandibular bone mass in Wnt1Tg mice after 1, 3, and 9 wk of *Wnt1* expression (Fig. 1D, upper panels). In addition, the alveolar ridges were higher in Wnt1Tg mice as compared to controls (Fig. 1D, lower panels). Quantification of the visible root area as well as mandibular bone volume revealed a significant bone growth already after 1 wk of *Wnt1* induction, which is in line with previous findings showing that *Wnt1* has a rapid bone-anabolic effect (Luther et al. 2018) (Fig. 1F, G). To our surprise, however, and contrary to these findings, we observed bone loss between the first and second molars in Wnt1Tg mice (Fig. 1E, red arrows). Although the variation of the observed alveolar bone loss was high, quantification of the distance between the cementum-enamel junction and the alveolar bone crest revealed significant differences between Wnt1Tg and control mice after 9 wk of *Wnt1* expression (Fig. 1H).

### *Wnt1* Overexpression Leads to Ectopic Calcification in the Pulp

**Figure 2.**
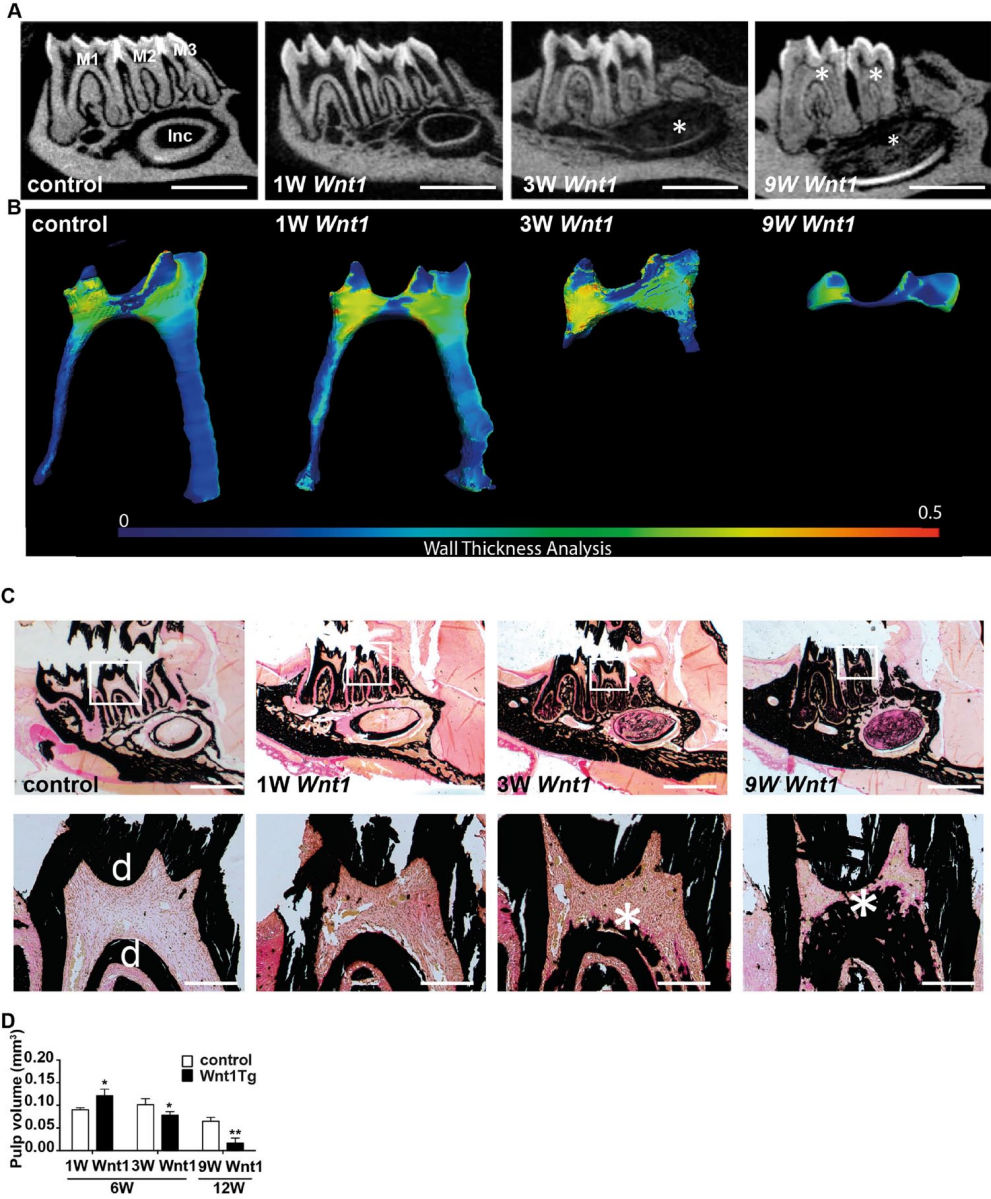
*Wnt1* causes ectopic calcification in the pulp. (**A**) Micro–computed tomography images of mandibular molars (M1–M3) from control and Wnt1Tg mice after 1 wk (1W Wnt1), 3 wk (3W Wnt1), and 9 wk (9 Wnt1) of *Wnt1* expression. Scale bars: 1 mm. (**B**) Wall thickness analysis of the first mandibular molar pulp from the same mice. (**C**) Undecalcified tooth sections of Wnt1Tg and control mice stained with von Kossa/van Gieson. Calcified tissue is stained black. Lower panels show higher magnifications of the regions marked by the white rectangles. Mineralized tissue (white asterisks) is present in the pulps after 3 and 9 wk of *Wnt1* expression. Scale bars: 1 mm (top panels), 0.1 mm (lower panels). (**D**) Quantification of pulp volume from wild-type (WT) and Wnt1Tg mice at 6 and 12 wk of age (*n* = 4–7). *P < 0.05.

We next investigated the mandibular teeth in more detail using micro-CT imaging as well as undecalcified histology (Fig. 2). We observed that tooth length was not different between Wnt1Tg mice and wild-type (WT) mice at all ages analyzed (Appendix Fig. 2A, B). However, wall thickness analysis of the pulp together with von Kossa/van Gieson staining of nondecalcified sections revealed a decrease in pulp volume and the presence of mineralized matrix within the pulp cavities of Wnt1Tg mice after 3 and 9 wk of *Wnt1* expression (Fig. 2A–D). Interestingly, our cellular analysis showed that the odontoblast layer was still present in incisors of Wnt1Tg mice after 1 wk of *Wnt1* expression (Appendix Fig. 3A, B). Interestingly, we observed in the pulp a significant increase in vascularization upon Wnt1 expression (Appendix Fig. 3C). Importantly, after 3 wk and 9 wk of *Wnt1* expression, the incisors were filled with ectopic calcifying matrix. A high proportion of this matrix consisted of nonmineralized organic tissue (45% after 3 wk of *Wnt1* expression and 40% after 9 wk of *Wnt1* expression) (Appendix Fig. 3D).

To further analyze the observed increase in calcified tissue in the pulp and the effect on dentin formation, we performed fluorescent imaging of calcein dye, which had been injected 10 and 3 d before sacrifice (Appendix Fig. 4). The calcein double layers indicated that dentin formation was still evident after 1 and 3 wk of *Wnt1* expression (Appendix Fig. 4B). This observation suggests that *Wnt1* expression in odontoblasts does not directly affect its function. In addition, the pattern of calcein labels in the pulp indicates that the ectopic formation of the calcified tissue begins at the root apices and moves coronally toward the pulp horns.

### *Wnt1* Overexpression Increases Cementum Formation

**Figure 3.**
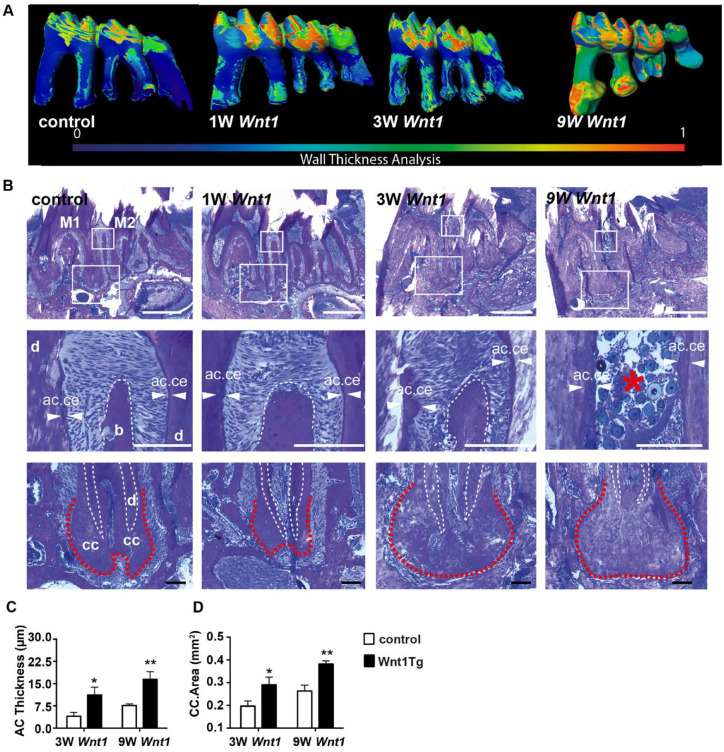
*Wnt1* increases acellular and cellular cementum formation. (**A**) Wall thickness analysis of mandibular molars from control and Wnt1Tg mice after 1 wk (1W Wnt1), 3 wk (3W Wnt1), and 9 wk (9 Wnt1) of *Wnt1* expression. (**B**) Undecalcified tooth sections of Wnt1Tg and control mice stained with toluidine blue. Middle and lower panels show higher magnifications of the regions marked by the white rectangles. Acellular cementum (ac.ce, white arrows) is present at the upper two-thirds of the root, whereas cellular cementum (cc, dashed red line) is present at the root apex. Acellular and cellular cementum is clearly thicker in Wnt1Tg mice with 3 and 9 wk of *Wnt1* expression as compared to those without. Note the loss of septal bone (dashed white lines, middle panels) associated with massive periodontal breakdown and food impaction (red asterisks) in Wnt1Tg mice after 9 wk of *Wnt1* expression. Scale bars: 0.5 mm (top panels), 100 µm (middle and lower panels). (**C**, **D**) Quantification of acellular cementum thickness (C) and cellular cementum area (D) of wild-type (WT) and WntTg mice. Six-week-old mice were deprived of doxycycline 3 wk before sacrifice, and 12-wk-old mice were deprived of doxycycline 9 wk before sacrifice (*n* = 3–5). *P < 0.05. **P < 0.001.

We next studied the root cementum in Wnt1Tg mice (Fig. 3). Micro-CT analyses together with histological examinations showed that 3 and 9 wk of *Wnt1* expression resulted in an increase of acellular and cellular cementum in Wnt1Tg mice (Fig. 3A, B). The acellular and cellular cementum layers were thicker after 3 and 9 wk of *Wnt1* expression as compared to that of mice without *Wnt1* expression (Fig. 3B). In fact, quantification revealed a 2-fold increase of acellular cementum thickness and cellular cementum area in Wnt1Tg mice after 3 and 9 wk of *Wnt1* expression as compared to control mice (Fig. 3C, D). In addition, the amount of nonmineralized cellular cementum (i.e., cementoid) was significantly higher in Wnt1Tg mice as compared to those of control mice (Appendix Fig. 5A, B). Interestingly, this increase of nonmineralized matrix was not evident in bone (Appendix Fig. 5C). Quantification of fluorescent calcein imaging further demonstrated high cementum formation after 3 wk of *Wnt1* expression, whereas no double calcein labels could be detected after 9 wk of *Wnt1* expression (Appendix Fig. 4C, D). However, an important histological finding was also the severe periodontal breakdown between the molar roots after 9 wk of *Wnt1* expression (Fig. 3B), confirming our micro-CT observations. In fact, von Kossa/van Gieson staining and polarized light imaging revealed detachment of PDL fibers in Wnt1Tg mice after 9 wk of *Wnt1* expression (Appendix Fig. 6). Taken together, these results suggest that *Wnt1* is a strong promoter of cementum formation. However, long-term exposure of *Wnt1* may induce periodontal destruction and ectopic calcification.

### The Ectopic Mineralization in the Pulp Has Cementum-Like Characteristics

**Figure 4.**
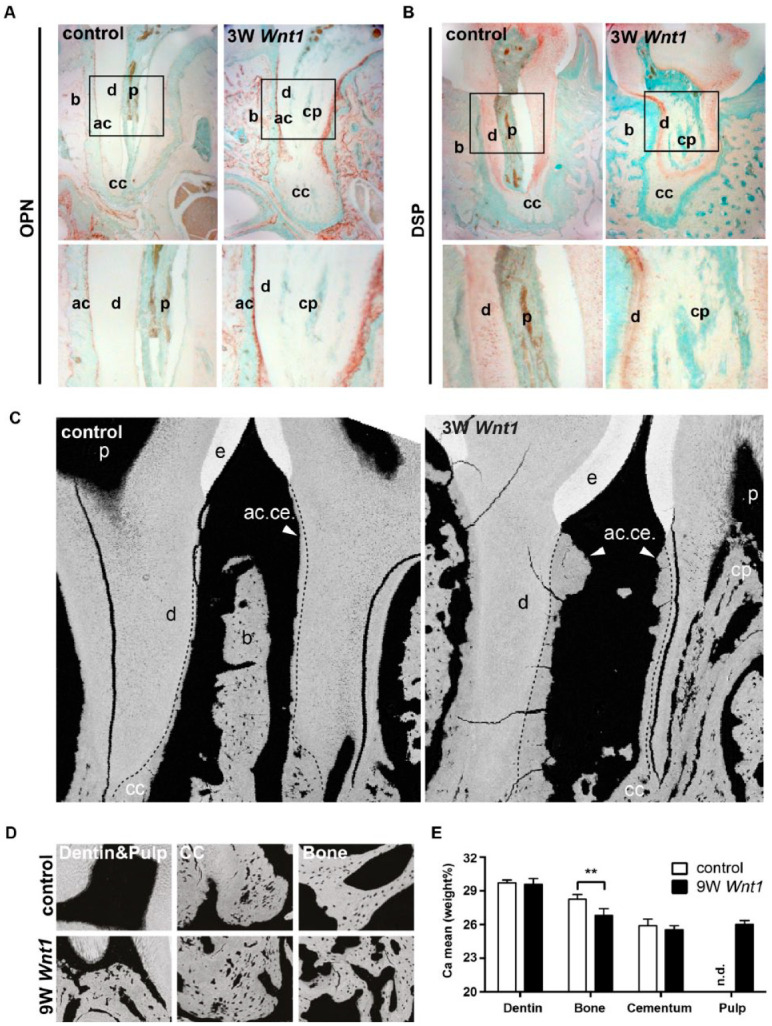
Characterization of ectopic pulp matrix in Wnt1Tg mice (**A**, **B**) Immunolocalization of osteopontin (A) and dentin sialophosphoprotein (B) in tooth sections of 6-wk-old wild-type and Wnt1Tg mice (3-wk *Wnt1* expression). Lower panels show higher magnifications of the regions marked by the black rectangles. ac, acellular cementum; b, bone; cc, cellular cementum; cp, calcified pulp; d, dentin. (**C**) Backscattered electron imaging of the periodontium from 12 wk-old wild-type and Wnt1Tg mice (9-wk *Wnt1* expression). ac.ce., acellular cementum; b, bone; cc, cellular cementum; cp, calcified pulp; e, enamel; p, pulp. (**D**) High-resolution backscattered electron images of dentin, bone, cementum, and pulp from the same mice. (**E**) Quantification of the mean mineral content (Ca mean) from each tissue (*n* = 4). *P < 0.01.

To further characterize the ectopic calcification, we used immunostaining for noncollagenous matrix proteins of Wnt1Tg and control mice (Fig. 4). Osteopontin (OPN) was expressed in bone and acellular cementum of Wnt1Tg and WT mice (Fig. 4A). The expression of OPN in these tissues was clearly higher in Wnt1Tg as compared to those of WT mice. Cellular cementum was only slightly stained for OPN, whereas no expression of OPN could be detected in the dentin layers. To further distinguish dentin from cementum, we next stained for the dentin-matrix protein dentin sialophosphoprotein (DSP). DSP was detected in the dentin layers of Wnt1Tg and WT mice (Fig. 4B). The calcified pulp tissue of Wnt1Tg mice showed only a slight expression of DSP. Almost no expression of DSP could be detected in bone and cementum in both mouse lines.

We next analyzed the calcium content using quantitative backscattered electron imaging and compared it with those of bone, dentin, and cementum (Fig. 4C–E). The backscattered images confirmed the periodontal bone loss and the increased cementum formation in Wnt1Tg (Fig. 4C). Quantification of the mean calcium content of dentin and cementum revealed no differences between Wnt1Tg and control mice (Fig. 4D, E). The mean calcium content of the alveolar bone was significantly lower in Wnt1Tg as compared to that of WT mice. This finding, however, is not unexpected given the fact that high bone formation can lower the mean calcium content as newly formed bone packages are less mineralized (Koehne et al. 2014). Importantly, the mean calcium content of the mineralized pulp matrix in Wnt1Tg was comparable to that of cellular cementum (Fig. 4E). These results altogether suggest that the calcium content and the distribution of noncollagenous proteins within the ectopic matrix share similarities with cellular cementum.

### *Wnt1* Overexpression Affects Cementoblast Proliferation Only in Growing Teeth

**Figure 5.**
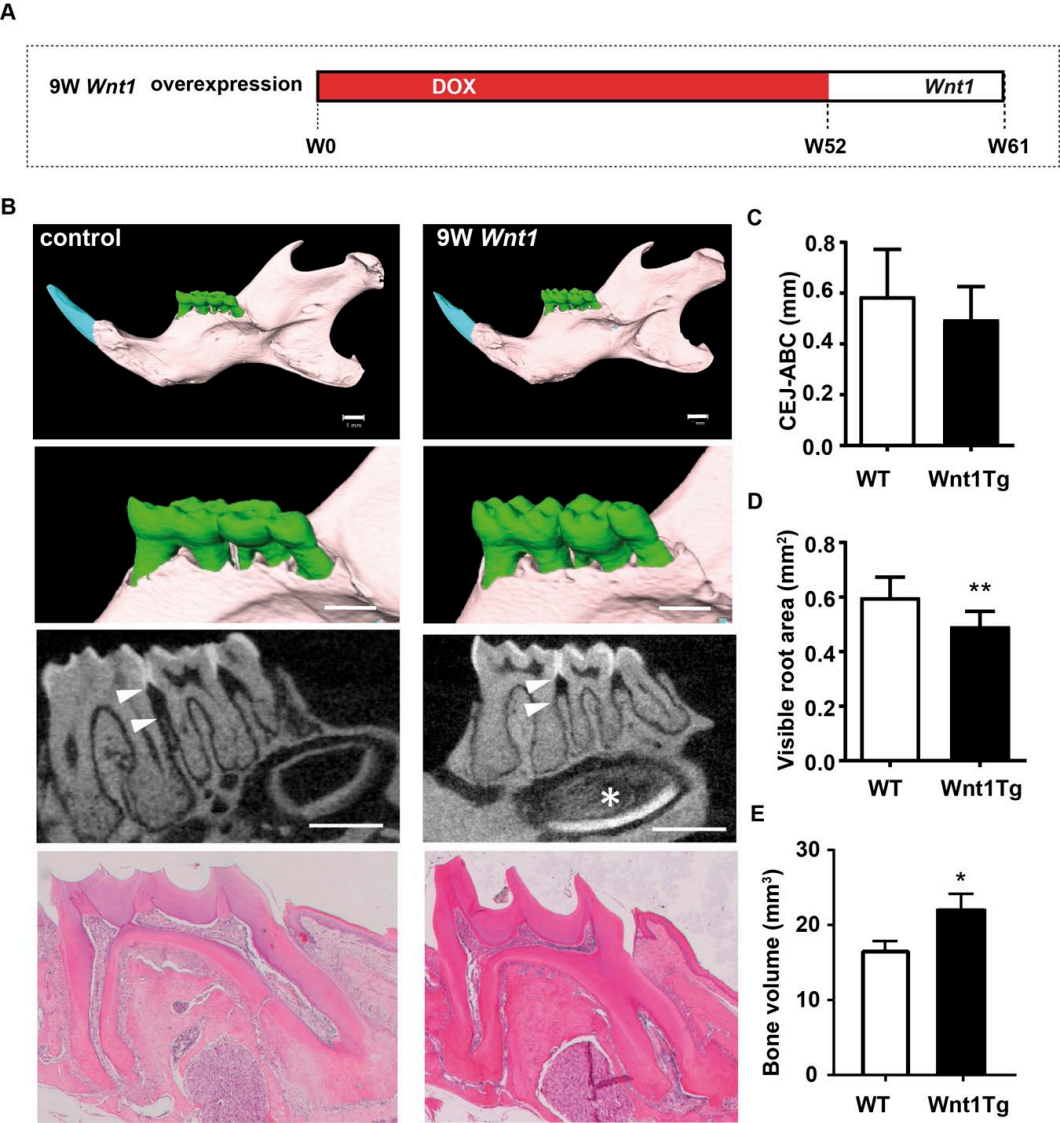
*Wnt1* expression affects only growing teeth. (**A**) Schematic representation of the feeding regimen. The 52-wk-old mice were deprived of doxycycline 9 wk before sacrifice to induce long-term expression of *Wnt1*. (**B**) Micro–computed tomography analysis of mandibles and mandibular molars from 52-wk-old Wnt1tg mice (9 wk of *Wnt1* expression) and controls. White triangles indicate the measured distance between the cemento-enamel junction (CEJ) and the alveolar bone crest (ABC). The pulp is fully calcified after 9 wk of *Wnt1* expression (white asterisk). Scale bars: 1 mm (upper and middle panel), 0.5 mm (lower panel). Decalcified molar section stained with hematoxylin-eosin revealed no differences between Wnt1tg and control mice. (**C–E**) Quantification of CEJ-ABC distance (C), visible root area (D), and mandibular bone volume (E) from wild-type (WT) and Wnt1Tg mice at 52 wk of age (*n* = 6–10). **P < 0.01.

We finally asked how *Wnt1* affects the periodontium of aged mice. To investigate this, we analyzed 52-wk-old mice after 9 wk of *Wnt1* expression (Fig. 5A). This experimental setup allowed us to study both developing teeth (incisor) as well as tooth maintenance (molar). Micro-CT imaging and decalcified histology revealed that the molars of aged Wnt1Tg mice were less affected by *Wnt1* induction (Fig. 5B). In fact, there was no evidence of pulp calcification or cementum increase. Furthermore, the periodontal bone between the first and second molars was not reduced in aged Wnt1Tg mice (Fig. 5C). The alveolar ridges, however, were higher in aged Wnt1Tg mice as compared to those of WT mice. Although this bone-anabolic effect was less clear as compared to those of younger Wnt1Tg mice, quantification of the visible root area revealed a significant reduction in Wnt1Tg mice as compared to WT (Fig. 5D). We also observed significantly increased mandibular bone volume in Wnt1Tg mice as compared to WT (Fig. 5E). This indicates that *Wnt1* promotes alveolar bone formation in aged mice without causing periodontal destruction as observed in younger mice. That *Wnt1* expression primarily affects growing teeth was further documented by the strong effect on the incisor, where the pulp appeared fully calcified (Fig. 5B).

Given these results, we finally determined the effect of *Wnt1* on cell proliferation. For this, we used the cementoblast cell line OCCM-30 stable expressing *Wnt1*-HA (Appendix Fig. 7A, B). Here we observed that *Wnt1* stimulates proliferation (Appendix Fig. 7C) but not differentiation of OCCM-30 cells (Appendix Fig. 7D, E).

Taken together, our results demonstrate that Wnt1 promotes cementum and alveolar bone growth. However, the exact molecular mechanisms of *Wnt1* on cementoblasts remain subject to further research.

## Discussion

Even though the role of WNT signaling in the regulation of tooth growth and maintenance is widely accepted (Tamura and Nemoto 2016), none of the 19 WNT ligands (except for *WNT10A* and *WNT10B*, which appeared to cause tooth agenesis) (Yu et al. 2016) have been identified to regulate tooth formation in vivo.

Here we present, for the first time in vivo, a novel role of *Wnt1* for cementum formation and periodontal homeostasis. More specifically, our histological analyses together with micro-CT imaging revealed that *Wnt1* is a strong promoter of alveolar bone and cementum formation. This is in line with a previous study showing that constitutive activation of β-catenin in the dental mesenchyme leads to differentiation of cementoblasts and induces excessive cementum formation in mice (Kim et al. 2011). However, by using an inducible and tissue-specific activation of WNT signaling, we provide several new insights in the role of WNT signaling for periodontal homeostasis. For example, one peculiar dental finding of this study was that long-term induction of *Wnt1* resulted in ectopic formation of calcified tissue within the pulp chambers. Backscattered imaging of the calcium content and immunostaining for noncollagenous protein revealed that the composition of this ectopic matrix features characteristics very similar to cellular cementum. This is in line with our in situ stainings showing expression of *Wnt1* in cementoblasts and cementocytes of cellular cementum. Our in vitro experiments using the OCCM-30 cementoblast cell lines further suggest that Wnt1 affects cementoblasts in a cell-autonomous manner. However, it is important to mention that our in vitro experiments do not entirely explain our findings in Wnt1tg mice, as we could not detect in OCCM-30 cells an increase in mineralization upon *Wnt1* overexpression. The precise mechanism of how *Wnt1* affects cementum formation, therefore, requires further investigation. Furthermore, we did not detect *Wnt1* expression in acellular cementum cementoblasts despite a clear increase of acellular cementum formation (Appendix Fig. 8). It, therefore, remains to be determined whether the pOBCol2.3 promoter used in this study to regulate *Wnt1* (Braut et al. 2002; Boban et al. 2006) is also expressed in acellular cementoblasts.

Furthermore, we show that *Wnt1* not only serves as a stimulant for cementoblasts but also regulates alveolar and mandibular bone formation. This confirms our previously published data on the bone-anabolic effect of *Wnt1* (Luther et al. 2018). Indeed, alveolar bone growth was evident in young as well as aged Wnt1tg mice. However, an unexpected finding in this regard was that long-term expression of *Wnt1* in young mice resulted in periodontal bone loss. We interpret this as a sign of overstimulation as cementum formation needs to be orchestrated with the formation of periodontal fibers to achieve proper periodontal anchorage. It, therefore, seems likely that long-term *Wnt1* expression causes an increase of dysfunctional cementum, in which PDL fibers cannot properly anchor anymore (Kim et al. 2011). This is further supported by our analyses of PDL organization in Wnt1tg mice. However, it is important to note that this periodontal breakdown was not observed in aged mice with long-term *Wnt1* induction. Although we did not investigate the expression pattern of *Wnt1* in aged mice, our analysis suggests that the detrimental effects of *Wnt1* might therefore be more relevant for growing teeth. In this regard, it might also be interesting to study vascularization processes during bone formation, which have not been analyzed in this mouse model so far, although WNT signaling is crucial for angiogenesis (Olsen et al. 2017). We observed increased vascularization in the dental pulp after 1 wk of *Wnt1* induction. Therefore, it seems that *Wnt1*-expressing odontoblasts induce angiogenesis by attracting endothelial cells (Masckauchan et al. 2005). This, however, has not been addressed in this study and deserves further experimental analysis.

Altogether, our findings open up a new paradigm in dental regeneration as β-catenin activating drugs such as BC21 (Kahn 2014) can have a huge effect on tooth development when given at a critical step during the development. Furthermore, prevention and management of alveolar bone loss are dependent upon regular periodontal tissue maintenance. Here we show that short-term induction of *Wnt1* can increase bone formation and therefore prevent alveolar bone loss while having a supporting effect on cementum formation. Nevertheless, long-term exposure will potentially lead to unwanted calcified pulp tissue as well as periodontal bone loss at least in growing teeth. This seems also important to consider when osteoanabolic medication harnessing the WNT pathway is given to young patients.

Taken together, our results provide new insights into the role of *Wnt1* for tooth development that can be employed for future regenerative strategies in dentistry.

## Author Contributions

C. Nottmeier, J. Petersen, T. Koehne, contributed to conception, design, data acquisition, analysis, and interpretation, drafted and critically revised the manuscript; N. Liao, contributed to conception, design, data acquisition, analysis, and interpretation, critically revised the manuscript; A. Simon, M.G. Decker, M. Kaucka, contributed to data acquisition, analysis, and interpretation, critically revised the manuscript; J. Luther, M. Schweizer, contributed to data acquisition and analysis, critically revised the manuscript; T. Yorgan, contributed to data acquisition and interpretation, critically revised the manuscript; E. Bockamp, contributed to data acquisition, critically revised the manuscript; B. Kahl-Nieke, M. Amling, T. Schinke, contributed to data interpretation, critically revised the manuscript. All authors gave final approval and agree to be accountable for all aspects of the work.

## Supplemental Material

sj-pdf-1-jdr-10.1177_00220345211012386 – Supplemental material for Wnt1 Promotes Cementum and Alveolar Bone Growth in a Time-Dependent Manner Click here for additional data file. Supplemental material, sj-pdf-1-jdr-10.1177_00220345211012386 for Wnt1 Promotes Cementum and Alveolar Bone Growth in a Time-Dependent Manner by C. Nottmeier, N. Liao, A. Simon, M.G. Decker, J. Luther, M. Schweizer, T. Yorgan, M. Kaucka, E. Bockamp, B. Kahl-Nieke, M. Amling, T. Schinke, J. Petersen and T. Koehne in Journal of Dental Research
