# Supplementary material for: Wnt1 Promotes Cementum and Alveolar Bone Growth in a Time-Dependent Manner
Source: J Dent Res. 2021 May 19;100(13):1501–9. doi: 10.1177/00220345211012386 (PMC8649456; doi:10.1177/00220345211012386)
Supplement: sj-pdf-1-jdr-10.1177_00220345211012386 – Supplemental material for Wnt1 Promotes Cementum and Alveolar Bone Growth in a Time-Dependent Manner [file sj-pdf-1-jdr-10.1177_00220345211012386.pdf]

Supplemental Appendix

Wnt1 promotes cementum and alveolar bone growth in a time-dependent manner

Authors: Cita Nottmeier[*1,2], Nannan Liao[1,4*], Alexander Simon[1], Maximilian G. Decker[1], Julia Luther[3], Michaela Schweizer[5], Timur Yorgan[3], Marketa Kaucka[6], Ernesto Bockamp[7], Bärbel Kahl-Nieke[1], Michael Amling[3], Thorsten Schinke[3], Julian Petersen[2,3§] and Till Koehne[1,2§]

Materials and Methods

Mice

All mice were kept in a specific pathogen-free environment with a 12-h light/dark cycle, 45-65% relative humidity, and 20-24 °C ambient temperature in open or individually ventilated cages with wood shavings bedding and nesting material in groups not surpassing 6 animals. The mice had access to tap water and standard rodent chow (1328P, Altromin Spezialfutter GmbH & Co. KG) or doxycycline-containing diet (A112-D00054, ssniff Spezialdiäten GmbH) ad libitum. All animal experiments were in accordance with the local implementation of the EU Directive 2010/63/EU and approved by the animal facility of the University Medical Center Hamburg-Eppendorf and the ethics committee (Behörde für Soziales, Familie, Gesundheit, und Verbraucherschutz) for animal experiments (G14/035, Org529, and Org869).

Micro-CT and stereomicroscopy

The skulls were scanned with a voxel resolution of 15 µm using micro-computed tomography (µCT 40, Scanco Medical, Switzerland) as described previously (Koehne et al. 2013). Visible root area and CEJ-ABC distance were analyzed on micro-CT images using ImageJ (Koehne et al. 2016; Nottmeier et al. 2020; Schneider et al. 2012). The pulp volume of the first mandibular molar was three-dimensionally determined using the MicroCT Software Suite 4.05 (Scanco Medical, Switzerland). Teeth and bone were segmented and analyzed using Avizo - 3D image data processing software (FEI, USA).

Histology

For decalcified histology, jaws were fixed in 3.5% PBS-buffered formaldehyde, dehydrated in ascending alcohol solution, and embedded in methylmethacrylate. 4-µm-thick sections were cut with a Microtec rotation microtome (Techno-Med, Germany) and stained by von Kossa/van Gieson, toluidine blue, and Masson's Trichrome staining according to standard protocols. Calcein double labels (injected 10 and 3 days before euthanization) were visualized with a

fluorescence microscope (Axioscope, Carl Zeiss Microscopy GmbH, Germany). Histomorphometry of cementum was performed using the OsteoMeasure system (Osteometrics Inc., USA) as previously described (Koehne et al. 2016). Briefly: Histomorphometric measurements were performed on toluidineblue–stained sagittal sections of the mandibular molars. Acellular cementum was quantified along its full length (500 to 700 µm) on the distal root surface of the first mandibular molar. Cellular cementum was measured at the distal root apex of the first mandibular molar. Calcein measurements were performed using the OsteoMeasure system (Osteometrics Inc., USA). Measurements of osteoid, cementoid, and the non-mineralized organic tissue within the ectopic calcified matrix were performed using OsteoidHisto according to the protocol (van 't Hof et al. 2017). Blood vessel areas were measured using ZEN Blue software and compared to the total pulp volume.

Immunohistochemistry

For immunohistochemistry, animals were deeply anesthetized by a mixture of ketanest and rompun and perfused through the heart with 4% paraformaldehyde (w/v) and 0.1% glutaraldehyde (w/v) in PBS.

The jaws were dissected sagittally and cryoprotected in 20% sucrose overnight. Thereafter, the tissue was embedded in optimal cutting temperature OCT compound (Tissue Tek O.C.T. Compound, Sakura) and frozen onto specimen holders at -20 °C. 16µm cryosections were prepared at -20 °C with and collected on super frost glass slides (CM 3050S from Leica).

After washing in phosphate-buffered saline (PBS) (pH 7,2), the sections were treated with 0.3% H_2O_2 and 1% $NaBH_4$ in PBS for 15 min to inhibit endogenous peroxidase activity. After rinsing in PBS, the sections were incubated with 10% horse serum containing 0.2% bovine serum albumin (BSA) (blocker) for 15 min to block nonspecific binding sites and incubated with the antibodies containing 1% horse serum and 0.2 % BSA (Carrier) overnight. The sections were washed with PBS, then incubated with biotinylated goat anti-rabbit IgG or goat anti-mouse IgG (Vector Laboratories, Burlingame, CA) diluted 1:1000 in Carrier for 90 min. After rinsing, they were incubated with ABC (Vector Labs) diluted 1:100 in PBS for 90 min. The sections were washed in PBS and reacted in diaminobenzidine (DAB)-H202 solution (Sigma St. Louis, USA) for 10 min. Sections were counterstained with methylene blue and embedded with Prolong Gold (Invitrogen).

Statistics

For each experiment including statistical analysis, a minimum of biological triplicates was used. Serial sections were allocated randomly to DOX-ON, DOX-OFF groups (coin flip), and

experiments (µCT and staining) were performed in a blinded fashion. Data are shown as means ± SEM

Fluorescent in situ hybridization (HCR 3.0)

Wnt1Tg and WT mouse skulls were fixed in 4% PFA (pH 7.4) overnight. Next, they were either decalcified in 10% EDTA for two weeks and dehydrated in 30% sucrose for 24 hours or embedded directly. Embedding was performed with OCT (Tissue Tek O.C.T. Compound, Sakura) and 10µm frozen tissue sections were cut using a Kryostat (CM 3050S from Leica). The fresh samples were washed with PBST 0,1% for 3x5min. The staining was performed, using the Molecular Instruments HCR v.3.0 protocol for „generic sample on slide“. The used probes (*Wnt1*, *Col1a1*) were designed and purchased from Molecular instruments (Los Angeles, CA 90041).

OCCM cells culture

The cementum cell line OCCM was cultured in Dulbecco's Modified Eagle Medium (DMEM + GlutaMAXTM-1) containing 10% Fetal Bovine Serum (FBS), and 1% Penicillin/Streptomycin at 37 °C in an atmosphere containing 5% CO2. The medium was changed every two days.

Stable transfection and selection

OCCM cells were transfected with the pLNCX control vector and the *Wnt1*-HA (Schulze et al. 2010) plasmid with Lipofectamine LTX and Plus reagent. After transfection, OCCM cells and cells were incubated for 1 day at 37 °C. After 24h, cells were split from 6-well plate into 10cm dishes and were put directly into the selective media (DMEM +/+, G418-BC: 10µl/ml). After 2-3 cell passages, the OCCM transfected cells were used in subsequent experiments.

Cell number determination

The OCCM cells were diluted to a suitable concentration. 10 µL OCCM cell suspension was pipetted in a Neubauer chamber. In order to accurately count the number, two out of 4 corner squares were employed to take the average cell count from each of the sets. The counted number was multiplied by 10,000 (10^4) and the dilution ratio. The unit is per milliliter.

Mineralization analysis

After 2 to 12 days of differentiation (induced with 50µg/mL Ascorbic acid and 10mM β-Glycerophosphate), cells were washed with 1x PBS, fixed with cold 90% ethanol for 10 minutes, washed twice with water, incubated with 40mM alizarin red staining solution at room

temperature for 10 minutes, and washed 3 times with water. After the cells were photographed on the lightbox, quantification was performed. For this, the stained cultures were incubated for 30 minutes in 800 µL 10 % acetic acid at room temperature, scraped off and pipetted into a 1.5 mL reaction tube, incubated for 5 minutes at 85°C and 2 minutes at 4 °C, and then centrifuged for 10 minutes at 13000xg. From the supernatant, 400 µL were carefully transferred to a new reaction tube and mixed with 50 µL 10% ammonium hydroxide solution. The absorbance was measured at 450nm.

Protein isolation

The transfected OCCM cells were lysed with 400 µL radio-immunoprecipitation assay buffer (RIPA buffer) per cell culture dish (⌀10), including protease and phosphatase inhibitors on day 5 of differentiation, scraped off and transferred to 1.5 mL reaction tube, incubated at 4 °C for 15 minutes, and then centrifuged for 15 minutes at 13000xg and 4 °C. The supernatant was pipetted into a new reaction tube, frozen in liquid nitrogen, and stored at - 80°C.

Western blot

Cell lysis was transferred from a SDS-PAGE gel (10%) onto a nitrocellulose membrane. The transfer process was performed using electroblotting at 4°C and 15V overnight. To examine the success of the transfer step, the total protein Ponceau S staining was performed. Subsequently, the nitrocellulose membrane was put into blotting buffer (5% BSA + 95% TBST) for 60 minutes at room temperature, transferred into primary antibody (HA-Tag, Cell Signaling, #2367 and β-Actin, Cell Signaling #4967) solution overnight at 4°C, washed 3 times with TBST (1X) for 5 minutes each time, transferred into secondary antibody solution for 60 minutes at room temperature, washed 3 times with TBST (1X) for 10 minutes each time, incubated in ECL western blotting solution for 1 minute and examined by western blot film in x-ray film processor. The exposure time varied between 30 seconds to 5 minutes.

Quantitative backscattered electron imaging

Methylmethacrylate-embedded specimens were polished planparallel and scanned with a backscattered electron microscope (LEO 435 VP; Leo Electron Microscopy Ltd., Cambridge, UK) at 20 kV and a 680-pA electron beam current as previously described (Koehne et al. 2014).

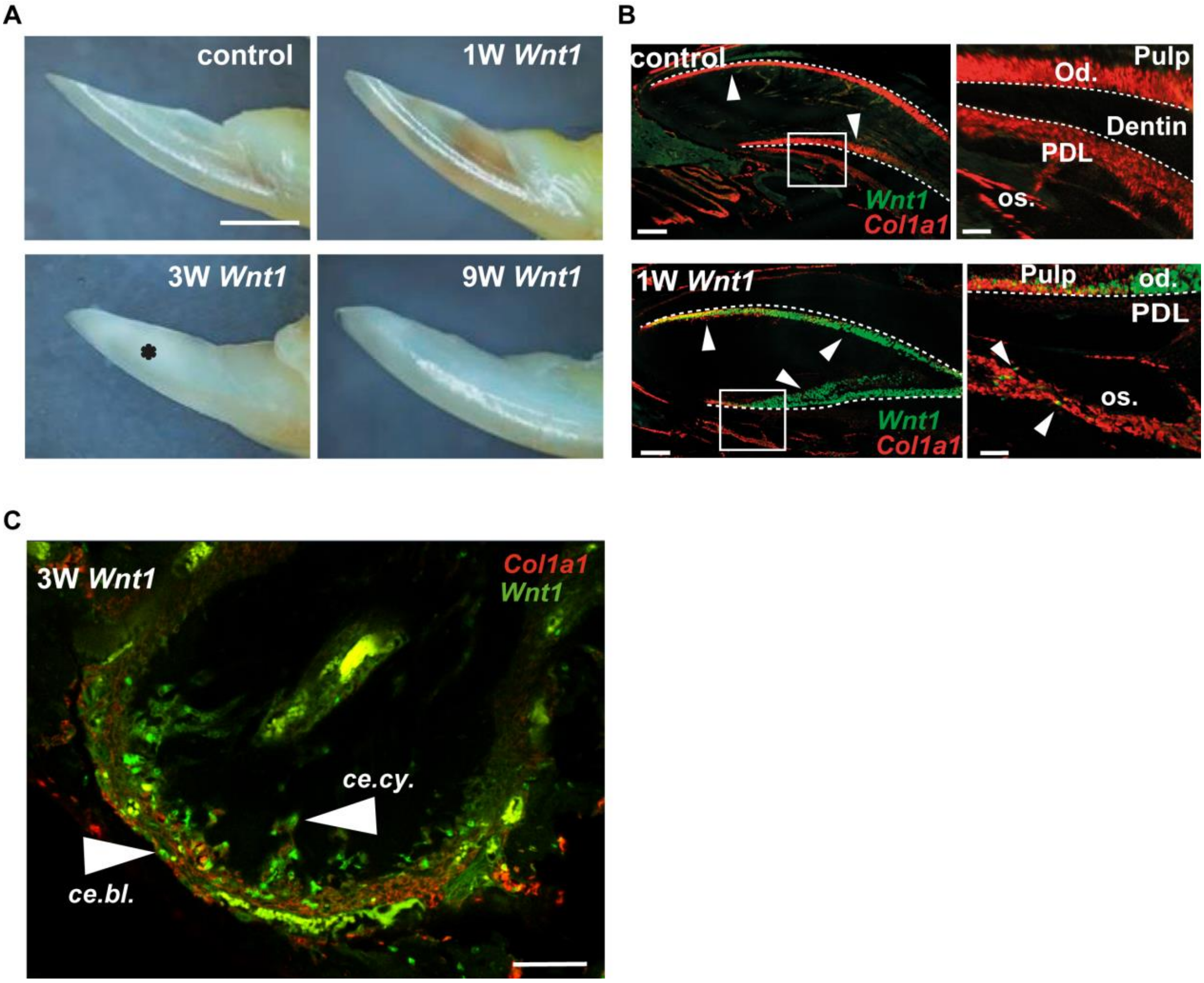

Supplementary Figure 1.

(**A**) Macroscopic images of mandibular incisors of wildtype (WT) and *Wnt1*-transgenic (Wnt1Tg) mice. *Wnt1* was induced for 1 week (1W *Wnt1*) and 3 weeks (3W *Wnt1*) in 6-weeks-old mice and 9 weeks (9W *Wnt1*) in 12-weeks-old mice. The incisors appear chalky white after 3 and 9 weeks of *Wnt1*-expression (asterisks). Scale bars = 1mm (**B**) Undecalcified in situ hybridizations stained for *Col1a1* and *Wnt1* mRNA of murine upper incisors from 6-weeks-old Wnt1Tg mice (1-week *Wnt1* expression) and control mice. os=osteoblasts, od=odontoblast, scale bar = 300µm (overview) and 50µm (enlarged section) (**C**) Undecalcified in situ hybridizations of molar roots from 6-weeks-old Wnt1Tg mice (3 weeks *Wnt1* expression) and control mice. ce.cy=cementocyte, ce.bl=cementoblast, Scale bar = 100µm.

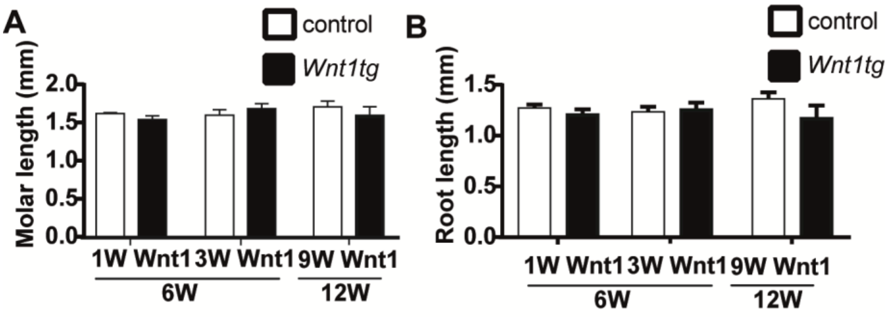

Supplementary Figure 2.

(A,B) Quantification of molar length (A) and root length (B) from WT and Wnt1Tg mice at 6 and 12 weeks of age (n=4-6).

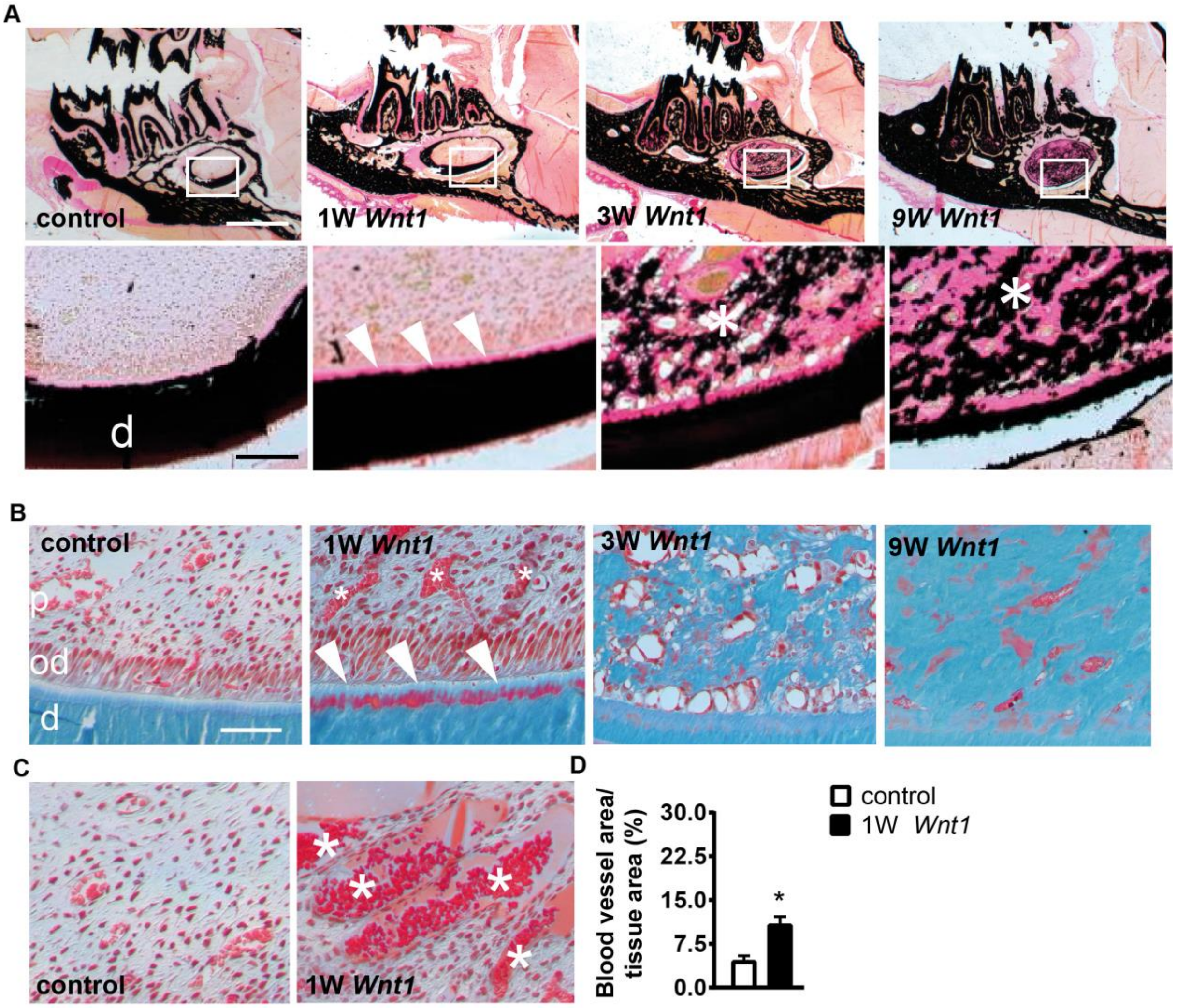

Supplementary Figure 3.

(A) Undecalcified tooth sections of Wnt1Tg mice stained with von Kossa/van Gieson. Wnt1Tg mice with 1, 3, and 9 weeks of *Wnt1*-expression were compared to mice without *Wnt1*-expression (control). Calcified tissue is stained black. Lower panels show higher magnifications of the regions marked by the white rectangles. Note the presence of non-mineralized predentin (white arrows) after 1 week of *Wnt1*-expression. Mineralized tissue (white asterisks) is present in the pulp cavities after 3 and 9 weeks *Wnt1*-expression. Scale bars = 1mm (top panels), 0.1 mm (lower panels). (**B**) Undecalcified incisor sections stained with Masson-Goldner trichrome. The odontoblasts (od) are normally aligned in Wnt1Tg mice without *Wnt1*-expression. One week of *Wnt1*-expression induces hypomineralization of the dentin (white arrows) and hypervascularisation of the pulp (p). No odontoblasts are evident after 3 and 9 weeks of *Wnt1*-expression. Scale bar = 50 µm. (**C-D**) Incisor pulp tissue of Wnt1Tg mice stained with Masson-Goldner trichrome. Numerous large blood vessels are present after 1 week of *Wnt1*-expression. Scale bar = 50 µm (n=3, *p<0.05).

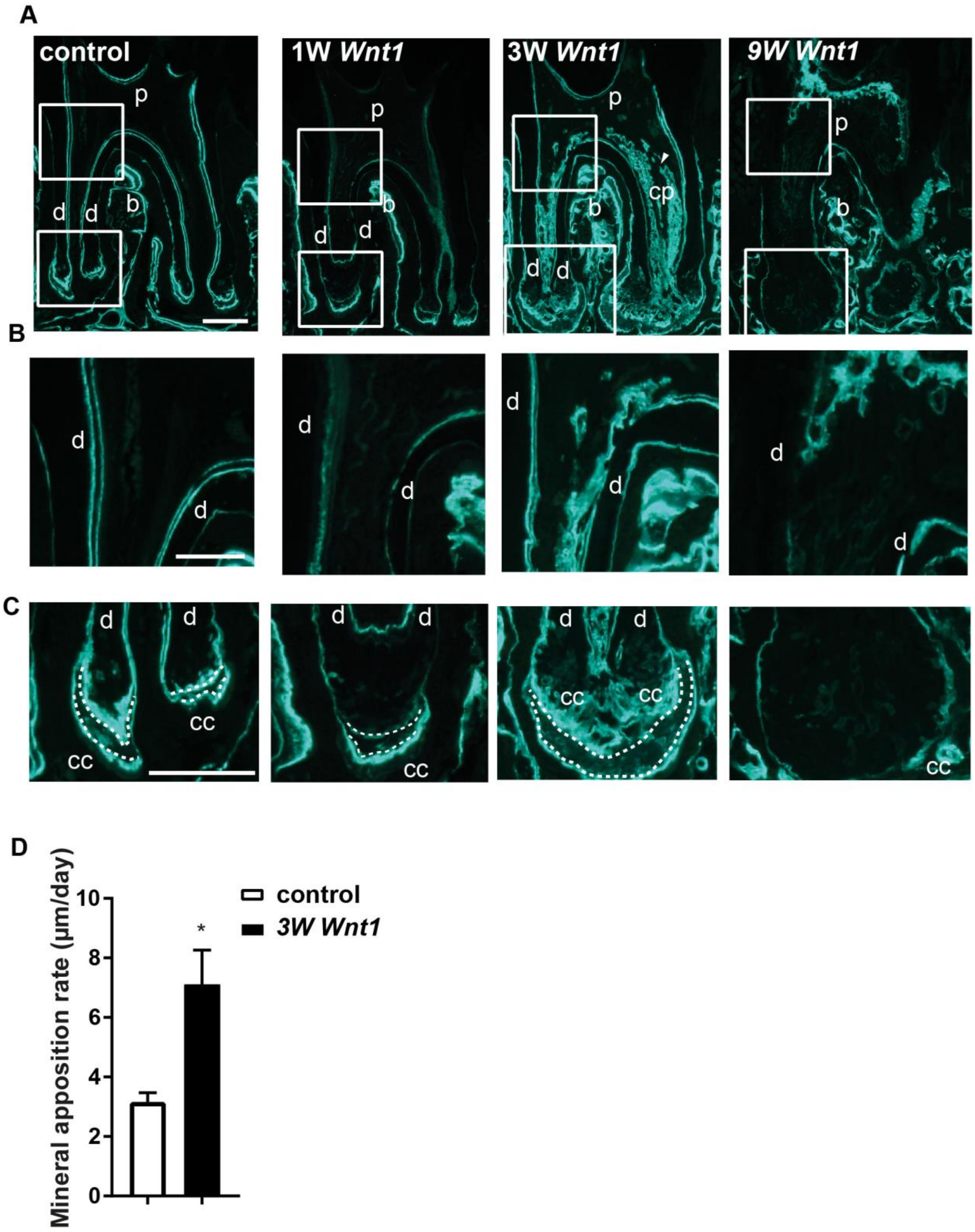

Supplementary Figure 4.

(A-C) Calcein fluorescent imaging of molars from Wnt1Tg and control mice. The regions marked by the white rectangles are shown at higher magnification below. The distance between the calcein double labels represents the amount of mineralized matrix formed within 1 week. Scale bars = 200 μm. p=pulp, b=bone, d=dentin, cc= cellular cementum, cp=calcified pulp **(D)**

Quantification of the mineral apposition rate for cellular cementum of 6-weeks-old Wnt1Tg (3 weeks of Wnt1 expression) and control mice (n=3, *$p<0.05$)

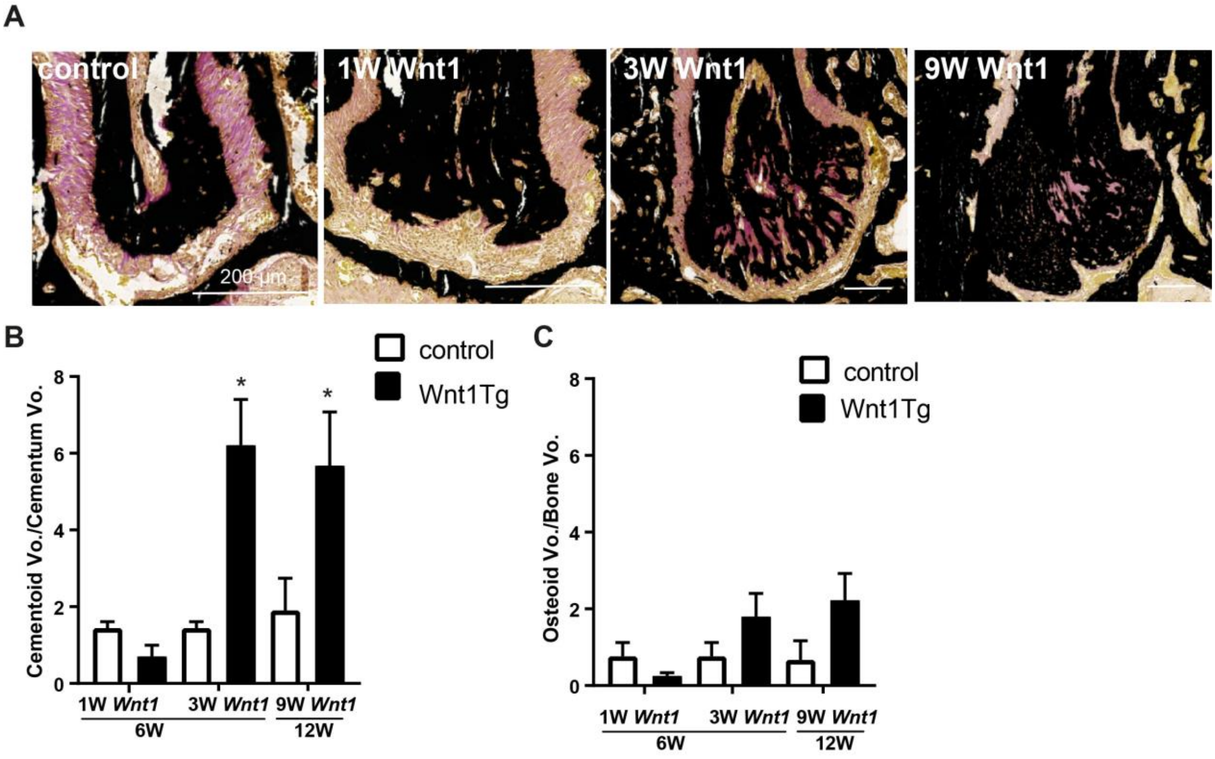

Supplementary Figure 5.

(**A**) Von Kossa/van Gieson staining of cellular cementum at the root apex of Wnt1Tg and control mice. (**B,C**) Quantification of the cementoid volume per cementum volume (B) and osteoid volume per bone volume (C) (n=3, *$p<0.05$).

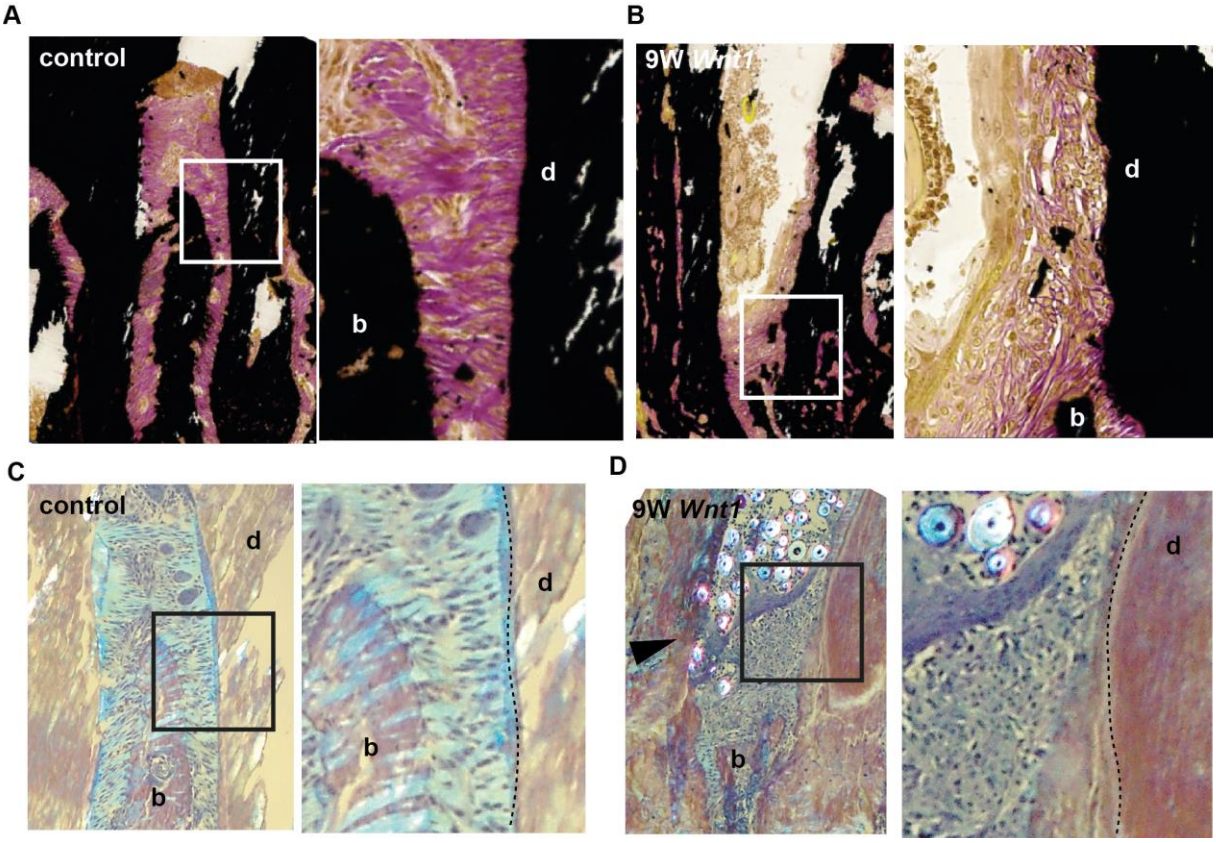

Supplementary Figure 6.

(**A-B**) Von Kossa/van Gieson staining of PDL fiber orientation between the 1st and 2nd mandibular molar of control (A) and Wnt1Tg (9W *Wnt1* expression) mice (B). (**C-D**) Polarized light microscopy of toluidine blue stainings from the same regions. Note the abnormal long junctional epithelium in Wnt1Tg mice (black arrow).

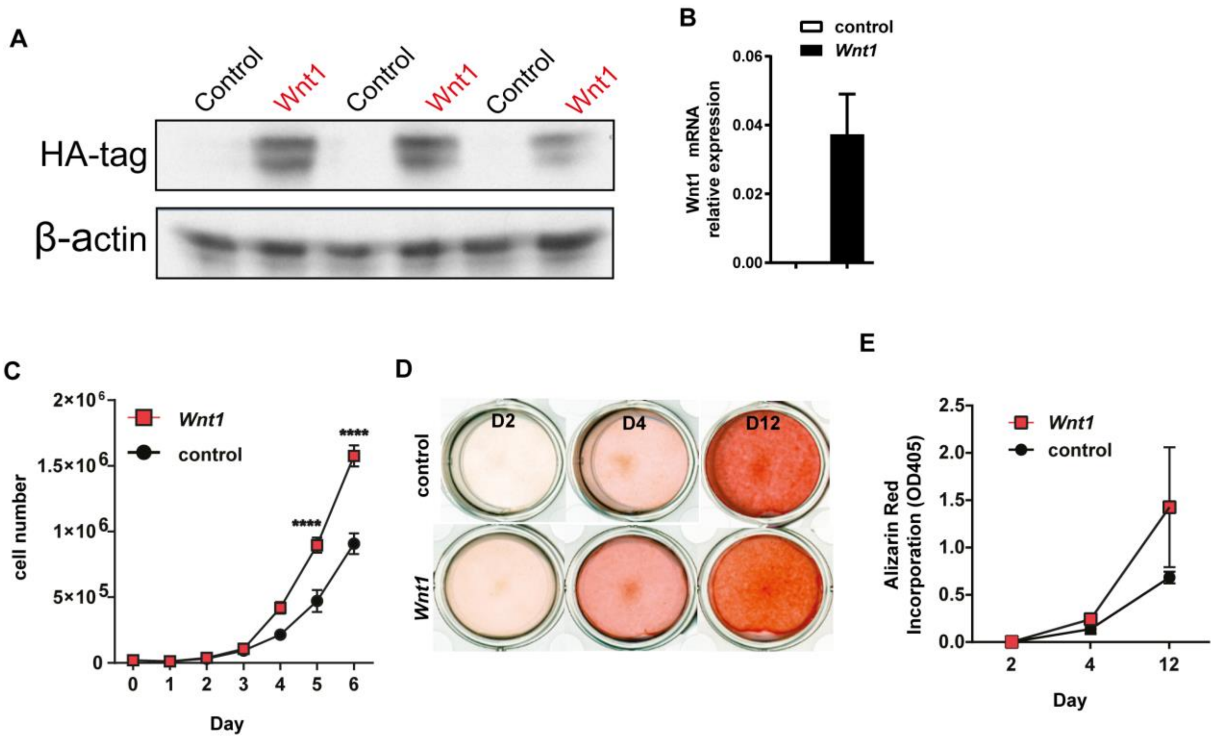

Supplementary Figure 7.

(A) Western blot analysis of *Wnt1*-expression using an HA-Tag antibody using protein extracts isolated from the 3 independently transfected OCCM cell cultures expressing *Wnt1* compared to vector-transfected controls. (**B**) Quantification of 3 independent western blot experiments Data are the means ± SEM. (**C**) Increased cell growth in 3 independent *Wnt1*-expressing OCCM cell cultures compared to vector-transfected controls. ****$p < 0.0001$ (**D)** Representative pictures of Alizarin Red staining and (**E**) quantification of Alizarin Red incorporation of 3 independent *Wnt1*-expressing OCCM cell cultures compared to control-transfected controls.

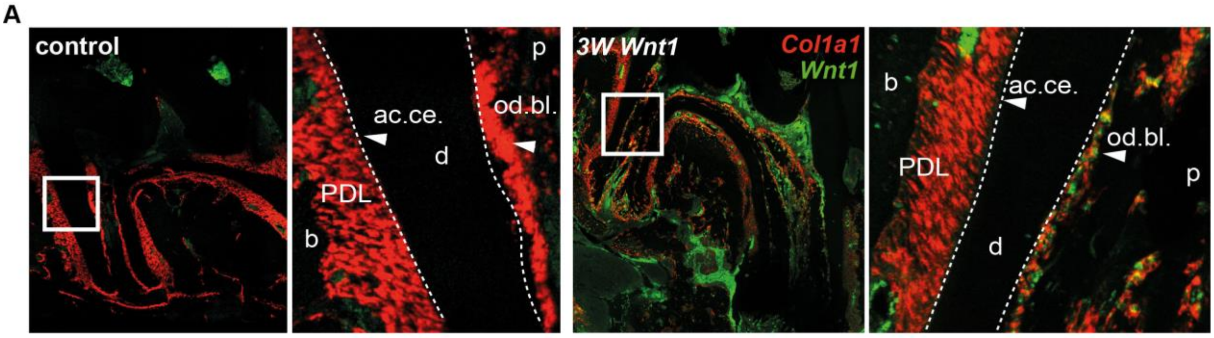

Supplementary Figure 8.

(A) Undecalcified in situ hybridizations of murine molars from control and Wnt1TG mice (3 W *Wnt1* expression) stained for *Col1a1* and *Wnt1* mRNA. Higher magnifications (white rectangles) demonstrate expression of *Wnt1* in odontoblasts, but not in cells of the acellular cementum. b=bone, p=pulp, d=dentin, ac.ce.=acellular cement, od.bl.=odontoblast.

References:

Koehne T, Jeschke A, Petermann F, Seitz S, Neven M, Peters S, Luther J, Schweizer M, Schinke T, Kahl-Nieke B et al. 2016. Rsk2, the kinase mutated in coffin-lowry syndrome, controls cementum formation. J Dent Res. 95(7):752-760.

Koehne T, Marshall RP, Jeschke A, Kahl-Nieke B, Schinke T, Amling M. 2013. Osteopetrosis, osteopetrorickets and hypophosphatemic rickets differentially affect dentin and enamel mineralization. Bone. 53(1):25-33.

Koehne T, Vettorazzi E, Kusters N, Luneburg R, Kahl-Nieke B, Puschel K, Amling M, Busse B. 2014. Trends in trabecular architecture and bone mineral density distribution in 152 individuals aged 30-90 years. Bone. 66:31-38.

Nottmeier C, Decker MG, Luther J, von Kroge S, Kahl-Nieke B, Amling M, Schinke T, Petersen J, Koehne T. 2020. Accelerated tooth movement in rsk2-deficient mice with impaired cementum formation. Int J Oral Sci. 12(1):35.

Schneider CA, Rasband WS, Eliceiri KW. 2012. Nih image to imagej: 25 years of image analysis. Nat Methods. 9(7):671-675.

Schulze J, Seitz S, Saito H, Schneebauer M, Marshall RP, Baranowsky A, Busse B, Schilling AF, Friedrich FW, Albers J et al. 2010. Negative regulation of bone formation by the transmembrane wnt antagonist kremen-2. PLoS One. 5(4):e10309.

van 't Hof RJ, Rose L, Bassonga E, Daroszewska A. 2017. Open source software for semi-automated histomorphometry of bone resorption and formation parameters. Bone. 99:69-79.
